# Nanodelivery Systems as New Tools for Immunostimulant or Vaccine Administration: Targeting the Fish Immune System

**DOI:** 10.3390/biology4040664

**Published:** 2015-10-19

**Authors:** Jie Ji, Debora Torrealba, Àngels Ruyra, Nerea Roher

**Affiliations:** Institut de Biotecnologia i de Biomedicina-Parc de Recerca UAB, Universitat Autònoma de Barcelona, Bellaterra 08193, Spain; E-Mails: Jie.Ji@uab.cat (J.J.); deboraalejandra.torrealba@uab.cat (D.T.); angelsruyra@gmail.com (A.R.)

**Keywords:** nanoparticles, fish, immunostimulation, alginate, liposome, chitosan, PLGA, carbon nanotubes

## Abstract

Fish disease treatments have progressed significantly over the last few years and have moved from the massive use of antibiotics to the development of vaccines mainly based on inactivated bacteria. Today, the incorporation of immunostimulants and antigens into nanomaterials provide us with new tools to enhance the performance of immunostimulation. Nanoparticles are dispersions or solid particles designed with specific physical properties (size, surface charge, or loading capacity), which allow controlled delivery and therefore improved targeting and stimulation of the immune system. The use of these nanodelivery platforms in fish is in the initial steps of development. Here we review the advances in the application of nanoparticles to fish disease prevention including: the type of biomaterial, the type of immunostimulant or vaccine loaded into the nanoparticles, and how they target the fish immune system.

## 1. Introduction

The use of vaccines has been essential in aquaculture and has been under development for over 70 years since the first successful fish vaccine was formulated [1]. Vaccines stimulate the immune system to mount a defence against a pathogen and as such to protect the host from infection by this pathogen. While they are extremely important to control infectious diseases in farmed fish, there are still some hurdles affecting the development of effective vaccines against viruses, parasites, and intracellular pathogens. One of these bottlenecks is the vaccine administration system [2,3]. Different approaches have been employed in aquaculture to improve the vaccine efficacy and to explore alternative routes of immunization. Traditional adjuvants such as mineral oils have been routinely used for vaccine injection, the most common examples are Freund’s complete adjuvant (FCA), Freund’s incomplete adjuvant (FIA) and more recently Montanide. Although adjuvants are very effective in potentiating the immune response against the pathogen, they present different side effects. There are three main methods for vaccine administration in fish: orally, by immersion or by injection. Vaccination by injection is the most reliable and effective system for vaccine delivery in fish and the protection is by far the most effective and long lasting [4,5]. However, the injectable vaccines are usually prepared with oil/water adjuvant formulations (FCA or FIA) which result in adverse effects such as the appearance of granulomas [2,6] adhesions between organ and peritoneal wall [7], injection site lesions [8,9], reduced appetite and growth [10], or deformations of the skeleton [11]. Added to this, the anaesthesia, handling, and injection may cause occasional mortality. Importantly, there are also logistical challenges to inject fish of less than 20 g in large numbers, but these fish need vaccination the most because they are the most susceptible to disease [12]. The disadvantages of vaccination by immersion are: the large amounts of vaccine required [13], the difficulty to measure the efficiency of the uptake [14] and the degradation of the compound in the water. Like for immersion, oral vaccines offer the advantages of being stress free and easy to administer to large numbers of fish but it is also difficult to determine the dose of antigen received. Also depending whether fish are gastric or agastric the intact antigen has to pass through the digestive system to reach the second segment of the hindgut where antigens are absorbed [15,16]. In this context, the use of nanodelivery systems has been proposed as an alternative strategy to address not only the above mentioned problems, but also to enhance the efficacy since some of these delivery systems may act also as a potent adjuvant, which is extremely important for anti-viral vaccines. Therefore, searching for new delivery systems is required to improve the administration and the efficacy of vaccines and immunostimulants.

Delivery systems are those materials used for the administration of pharmaceuticals in a controlled manner aimed to achieve a therapeutic effect. These systems provide: cell or tissue targeted delivery of active compounds, improved bioavailability, improved solubility of hydrophobic drugs, sustained release and protection of the therapeutic agent from degradation [17]. Nanoencapsulation involves forming drug loaded particles with diameters ranging from 1 to 1000 nm, although other stricter definitions refer only to structures in the 1–100 nm range (*US National Nanotechnology Initiative, What is nanotechnology?*). This size property enables the nanoscale devices to readily interact with biomolecules, such as enzymes and receptors, both on the surface and inside the cells.

Since 1960, when the first liposomes for drug delivery were described, a variety of other organic and inorganic biomaterials were developed for drug delivery [17]. In 1980 more complex drug delivery systems capable of responding to pH changes to trigger drug release or the first examples of cell specific targeting of liposomes were described [18]. Nowadays, nanoparticles can be easily tuned to have unique physical characteristics in size, shape, surface chemistry, or targeted surface ligand/receptor. The benefits of nanoparticles as delivery tools are the reduction of the doses, tissue specific targeting, reduction of the toxic or secondary effects of the drug and increase in the delivery efficiency [19]. The encapsulated molecules will generally have completely different properties (e.g., solubility or circulating half-life) compared to the non-encapsulated ones. Thus, it is very important to understand and control the *in vivo* behaviour on cells or tissues of these bioactive compounds once encapsulated, to know their efficacy and side effects. As mentioned, the size of the nanoparticle is not only important for the interaction with biomolecules but also because it will influence its biodistribution *in vivo*. In mammals it has been extensively studied that particles of less than 5 nm are cleared from the circulation through extravasation or renal clearance, whereas bigger nanoparticles (up to 15 μm) accumulate in the spleen, liver, and bone marrow [20]. The particle size also influences the preferred mechanism of cellular internalization, such as phagocytosis, macropinocytosis, caveolae-mediated-endocytosis, or others. Of note, the fate of the internalized material will be different in each case [21,22].

In teleosts, there are few reports on how the nanoparticles spread throughout the organism through the circulatory system, gills, gut, spleen, liver, or brain depending on the administration route [23,24]. Current findings indicate that particle shape and rigidity are also key factors for the kinetics and fate of the nanoparticles, mainly affecting the endocytosis. The vast majority of nanoparticles have a spherical shape, however similar volumes with different shapes are internalized at different rates [25,26]. Increased nanoparticle rigidity is related to enhanced phagocytosis by macrophages [27]. Finally, the nanoparticle surface charge critically affects how they interact with serum proteins and cell membranes. Highly charged particles fix more complement proteins [28], a process that can only be inhibited by addition of a hydrophilic coating. The surface charge will also determine the interaction with cell membranes. In general, neutral and anionic nanoparticles will be less internalized than positively charged ones [22,29]. Different studies using the same nanoparticle with different surface charges have shown that those with cationic groups were internalized more efficiently, [30,31] mostly due to the high affinity for the negatively charged proteoglycans present on the surface of cells [32]. The use of nanoparticles does also have some limitations. For example, their small size and large surface area can lead to particle aggregation and result in limited drug loading and burst release, making physical handling of nanoparticles difficult in liquid and dry forms [33]. Another issue that should be addressed in the future is the safety, for both human and animals, not only of the delivery system itself but also of the degradation products of the nanoparticles. These biosafety issues should be carefully addressed to avoid environmental contamination that can provoke detrimental effects on animal and human health.

In this review, we summarized different nano- and micro-sized delivery systems that have been described as delivery tools for fish vaccination or immunostimulation. Calcium phosphate nanoparticles, carbon nanotubes, chitosan nanoparticles, liposomes, poly-lactic-glycolic acid nanoparticles, or alginate micro-particles are described in detail below.

## 2. Nanodelivery Systems

### 2.1. Alginate

Alginate is found naturally in brown algae, such as *Laminaria hyperborea*, *Laminaria digitata*, *Macrocystes pyrifera*, and *Lessonia nigrescens*. It can also be found as a polysaccharide in some bacteria such as *Azotobacter vinelandii* and *Pseudomonas* [34]. Alginate is a generic name used to define a complex molecule made of repeated units of the unbranched polyanionic polysaccharides α-l-guluronic acid (G) and β-d-mannuronic acid (M). Alginate is built by combination of G-G, G-M, and/or M-M blocks. These blocks can be found in different G/M composition and chain arrangements, which gives them its differential physico-chemical properties [35,36]. The mechanical and the physical stability of alginate mainly depend on the G content, the greater the G content, the more rigid and brittle the matrix [37]. Alginate-microparticles (alginate-MPs) are eroded at neutral and basic pH allowing the release of the cargo by diffusion, while at low pH values they are extremely stable [38]. This stability at low pH makes alginate-MPs suitable for oral administration, since in the fish stomach (pH between 2 and 4) the release will be low while the release in the foregut or hindgut at neutral-basic pH (pH 7 and 8.3, respectively) will be high [39,40]. Notably, alginate is mucoadhesive allowing the adhesion to the epithelial mucus (e.g., intestinal mucosa) and making it very attractive for oral administration. Other important features of alginate-MPs are the high biocompatibility and the low cost of production.

Alginate-MPs can be produced by classical techniques such as air atomization, emulsification, gelation, and complexation with counterion polymers, or by new methods, such as spray-drying, electrohydrodynamic atomization, impinging aerosols, and inkjet/drying process, that enable a better control of the size [37]. For application in fish, alginate-MPs are generally produced by emulsification [39,40,41,42] that is one of the fastest methods for nanoparticle preparation and is readily scalable [43], and to a lesser extent by other methodologies such as the orifice-ionic gelation and the spray method [44,45] (Table 1).

In mammals, alginate nanoparticles have been used for the delivery of different drugs [46,47,48,49], but to date there are no alginate nano-sized particles routinely used for delivery of active compounds in fish. Nevertheless, micro-sized alginate particles are one of the most common delivery systems assayed in fish with promising results for viral diseases.

#### 2.1.1. Encapsulation of Bacterial Antigens in Alginate Microparticles

To date, the main bacterial antigens encapsulated into alginate-MPs have been formalin-killed bacteria (FKB) from different species (Table 1). FKB have been widely used as antigens for fish vaccination in some diseases, mainly those caused by Gram-negative bacteria. In general, FKB vaccines provide excellent levels of protection by itself or in combination with an adjuvant (e.g., FCA) [40,50]. In general, oral administration of FKB encapsulated in alginate-MPs does not work very well alone, and only when combining the alginate-MPs with the FKB vaccine they obtained a longer lasting protection. The oral administration of alginate-FKB from *Lactococcus garviaeae* in rainbow trout (*Oncorhynchus mykiss*) provided low levels of protection against *L. garviaeae* infection (35% Relative percent survival (RPS) at 30 days) compared with the naked FKB vaccine administrated intraperitoneally (100% RPS at 30 days and 40% at 90 days) (Table 1). These results were improved when fish were immunized orally a second time with the alginate-vaccine three months later (61% RPS at 180 days) [44]. Altun and coworkers observed similar result with this alginate-construct administrated orally in rainbow trout (Table 1). It did not provide better protection against *L. garviaeae* infection (53% RPS at 30 days and 38% RPS at 60 days) than the naked vaccine (95% RPS at 30 days and 82% RPS at 60 days). The protection was again increased when fish was immunized a second time with alginate-FKB-LG at day 61 (67% RPS at 90 days and 62% RPS at 120 days) or with a first administration of naked vaccine and then a second administration of the alginate-construct at day 61 (86% RPS at 90 days and 81% RPS at 120 days) [45].

Leal *et al.* [40] evaluated the alginate-MPs formulated with FKB from *Flavobacterium columnare* in nile tilapia (*Oreochromis niloticus*) (Table 1). Alginate-vaccine and naked vaccine administrated orally did not provide protection against *F. columnare* challenge (0% of RPS at 21 days in both cases) and did not stimulate the production of specific antibodies against *F. columnare* in immunized fish [40].

#### 2.1.2. Encapsulation of Viral DNA in Alginate Microparticles

For viral diseases, alginate-MPs have been used to encapsulate DNA vaccines made with plasmids coding for viral proteins. The alginate-MPs loaded with DNA vaccines are smaller (≤ 10 µm) than the alginate-MPs loaded with bacterial antigens (10–30 µm) [44] and this seems to favor the targeting of different organs, such as spleen, kidney, liver, pyloric caeca, heart, intestine, or gills [41,42]. Alginate-MPs containing the plasmid coding for the major capsid protein (MCP) of Lymphocystis Disease Virus (LCDV) increased the titer of specific antibodies against LCDV in olive flounder (*Paralichthys olivaceus*) serum after oral administration (Table 1). The results showed a progressive increase until week 11 while the naked DNA vaccine did not stimulate any increase in the antibody titer. The naked DNA vaccine might thus be hydrolyzed in the gastrointestinal tract while the alginate-MPs can reach the tissues [41].

Alginate-MPs with a plasmid coding for VP2, one of the major structural proteins of Infectious Pancreatic Necrosis Virus (IPNV) stimulated the production of specific neutralizing antibodies in *O. mykiss* until eight weeks after oral administration (Table 1). In infection experiments with this virus, alginate-MPs orally administrated to *O. mykiss* and *Salmo trutta* increased the protection levels nearly to 80% RPS at 15 and 30 days post-vaccination [42]. These levels of protection were comparable with a commercial subunit vaccine (e.g., Microtek) administrated by intraperitoneal injection [51].

**Table 1:** Microparticles used as delivery systems in fish.

Microparticle	Size	Production Technique and Composition	Encapsulated Molecule	Administration	Species	Fish Size	RPS	Reference
Alginate	30 µm	Spray method, sodium alginate, 0.5% (*w/v*)	FKB from *Lactococcus garviaeae*	Oral	*Onchorhynchus mykiss*	22 g	35% E and 100% N at 30 DPV; 5% E and 40% N at 90 DPV; 61% first V with N and second with E at 180 DPV	[44]
	n.d.	Orifice-ionic gelation, Sodium alginate, 4% (*w/v*)	FKB From *Lactococcus garviaeae*	Oral	*Onchorhynchus mykiss*	20 g	53% E and 95% N at 30 DPV; 38% E and 82% at 60 DPV; 67% first V with N and second with E at 90 DPV; 62% first and second V with E at 120 DPV	[45]
	n.d.	Emulsification, sodium alginate, 3.5% (*w/v*)	FKB from *Flavobacterium columnare*	Oral	*Oreochromis niloticus*	15.7 g	0% E and 0% N at 21 DPC	[40]
	≤ 10 µm	Emulsification, sodium alginate, 3% (*w/v*)	Plasmid DNA: MCP from LCDV	Oral	*Paralichthys olivaceus*	40–60 g	n.d.	[41]
	10 µm	Emulsification, sodium alginate, 3% (*w/v*)	Plasmid DNA: VP2 from IPNV	Oral	*Salmo trutta*	1.5 g/3 cm	At 15 DPV: 78% E and 0% empty plasmid at 30 DPC, At 30 DPV: 79% and 0% empty plasmid at 30 DPC (*)	[42]
	10 µm	Emulsification, sodium alginate, 3% (*w/v*)	Plasmid DNA: VP2 from IPNV	Oral	*Onchorhynchus mykiss*	1 g/3.5 cm	At 15 DPV: 80% E and 5% empty plasmid at 30 DPC; At 30 DPV: 67% and 0% empty plasmid at 30 DPC (*)	[42]
Chitosan	≤ 10 µm	Emulsification, 3% chitosan (*m/v*)	Plasmid DNA: MCP from LCDV	Oral	*Paralichthys olivaceus*	50–100 g and 13–15 cm	n.d.	[52]
	< 5 µm	Spray drying, 240 mg of PVMMA and 250 mg of chitosan Seacure 210 HCl	Surface antigens (Ag) from *Philasterides dicentrarchi*	i.p. injection	*Scophthalmus maximus*	50 g	68% E, 58% Ag in FCA and 43% FCA at 20 DPC	[53]
	4.28 ± 0.4 µm	Spray drying, 240 mg of GantrezAN119 and 250 mg of chitosan Seacure 210 HCl	Surface antigens (Ag) from *Philasterides dicentrarchi*	*In vitro*, anterior kidney leukocytes	*Scophthalmus maximus*	n.d	n.d.	[54]
	1.101 ± 0.0103 µm	TPP ionic gelation, 5 mg/mL chitosan in sodium alginate solution at concentration of 10 mg/mL	FKB from *Aeromonas hydrophila*	Oral	*Labeo rohita*	Juveniles	13% alginate and chitosan E, 13% chitosan E, 16% alginate and chitosan, 0% N at 15 DPC (*)	[55]
PLGA	1.12 µm	D.E., PLGA 50:50, MW: 30–70 kDa	OMP from *Aeromona hydrophila*	Parenteral	*Labeo rohita*	30–40 g and 250–300 g	n.d.	[56]
	< 10 µm	D.E., L:G = 75:25, MW:50 kDa	Plasmid DNA: MCP from LCDV	Oral	*Paralichthys olivaceus*	500–1000 g	n.d.	[57]
	1 µm	Emulsion, PLGA 50:50	γ-globulins from human blood	Oral	*Onchorhynchus mykiss*	100–200 g	n.d.	[58]
	n.d.	D.E., PLGA 50:50	i-antigen from *Uromena marinum*	i.p. injection	*Epinephelus bruneus*	31.4 ± 2.3 g	78% E and 66% N at 30 DPC (*)	[59]
PLGA/Liposome	5–10 µm	Film dispersion method, PS, PC, and Chol (molar ratio 1:10:5)	FKB from *Aeromonas hydrophila*	Oral	*Cyprinus carpio*	30 g	64% E at 12 DPC	[60]
	n.d.	D.E., PLGA 50:50	ODN1668	i.p. injection	*Epinephelus bruneus*	36.7 ± 2.8 g	78% PLGA E, 83% Liposome E, 83% PLGA/Liposome E and 78% N at 30 DPC (*)	[61]

Chol: Cholesterol; D.E.: double emulsion; DPC: days post-challenge; DPV: days post-vaccination; E.: encapsulated antigen; FCA: Freund’s complete adjuvant; FKB: formalin killed bacteria; GantrezAN119: methyl vinyl ether-co-maleic anhydride; i.p.: intraperitoneal; IPNV: Infectious pancreatic necrosis virus; MCP: major capsid protein; N: naked antigen; n.d.: not described; LCDV: Lymphocystis disease virus; ODN1668: oligodeoxynucleotide 1668; Omp: outer membrane protein; PC: Phosphatidylcholine; PLGA: poly(lactic-co-glycolic acid); PS: Phosphatidylserine; PVMMA: Poly (methyl vinyl ether)-co-(maleic anhydre); RPS: Relative percent survival; V: vaccination; VP2: Viral protein 2; (*): calculated RPS.

### 2.2. Carbon nanotubes

Carbon nanotubes (CNTs) were discovered in 1991 by Iijima [62]. CNTs are allotropes of carbon with a cylindrical nanostructure and this network of carbon atoms can reach several micrometers in length with a nanosized diameter. CNTs can be produced at large scale by three methods: discharge, laser ablation, and chemical vapor deposition. During the production process with all these methods impurities are formed, thus requiring an additional purification step [63]. Pure CNTs are not soluble in aqueous solutions because they have highly hydrophobic surfaces and an additional functionalization step is needed. There are two main types of carbon nanotubes, single-walled, and multi-walled. Single-walled CNTs are flexible but require catalytic synthesis making its bulk production difficult and leading to poor levels of purity. Multi-walled CNTs are formed by several concentric layers and thus are more rigid. They can be produced without catalyst, which allows bulk synthesis and high purity [64]. CNTs are chemically stable, relatively inert, non-immunogenic, and non-toxic. Additionally, CNTs have a large surface area available and are able to absorb or to be conjugated to a wide variety of antigens, presenting high stability *in vivo* [65,66]. In mammals, CNTs are being investigated as a delivery system for genes, peptides, oligonucleotides, antimicrobial agents, and cytotoxic drugs [67,68,69,70]. In fish, the study of CNTs as delivery systems has recently started, focusing on its functionalization with chemical groups and proteins and on the encapsulation of DNA vaccines [71,72,73] (Table 2).

#### 2.2.1. Functionalization of CNTs

As mentioned above, functionalization is required to solubilize the CNTs and to make them biocompatible. This process can be divided in two different approaches, depending on the covalent/non-covalent nature of the linked antigens [64]. The covalent attachment of different chemical groups (e.g., sulfonate) and proteins (e.g., bovine serum albumin) has been used to design nanoparticles for fish [71,72,73]. Different studies warn about the potential for these manufactured nanomaterials to contaminate the aquatic environment. To evaluate immunotoxicity, functionalized single-walled and multi-walled CTNs with chemical groups, such as sulfonate, sulfonic acid, and polyethylene glycol were tested for toxicity in head kidney macrophages isolated from *O. mykiss* (Table 2)*.* The CNTs formulations did not decrease the cell viability after 24 h treatment [72]. None of these formulations stimulated the expression of interferon alpha (IFNα) gene, however CNTs with and without functionalization stimulated interleukin 1 beta (IL-1β) gene expression in trout macrophages indicating that they can be pro-inflammatory if they gain entry to the body. Multi-walled CNTs containing anionic groups (sulfonate groups) caused the highest IL-1β stimulation, while single-walled CNTs containing neutral groups (polyethylene glycol groups) caused the least reaction. The functionalized CNTs were also more potent in stimulating gene expression than the non-functionalized counterparts [72].

The functionalized CNTs thus produce a stimulation of the immune system by themselves without any loaded antigen [71,72], although there is no information about the levels of protection that they may provide in a challenge. Fluorescent multi-walled CNTs functionalized with bovine serum albumin (BSA) were tested in zebrafish (*Danio rerio*) embryos by microinjection into the circulation at 72 h post fertilization (Table 2). These CNTs distributed all along the blood circulation and then moved to the muscle, brain ventricle and notochord, being finally cleared out at 96 h after injection. The immune response of the embryos was studied by *in situ* hybridization of Matrix Metalloproteinase 9 (MMP9). At early stages, the injected embryos showed an increase in MMP9 expression levels and changes in the expression pattern. These results suggest that embryos may generate an innate immune response when being injected with CNTs at the 1-cell stage. The injected zebrafish embryos had normal primordial germ cells and were able to produce a new generation at the adult stage. However, the larvae of the second generation showed lower survival rates as compared with the untreated group, suggesting a negative effect on the reproduction potential [71].

#### 2.2.2. Encapsulation of Viral DNA in CNTs

To date only one work has evaluated CNTs as a DNA delivery system in fish, but with promising results. Single-walled CNTs were loaded with a plasmid encoding the VP7 protein of Grass Carp Reovirus (GCRV). The plasmid expression after intramuscular injection in grass carp (*Ctenopharyngodon idella*) was detected at high levels in muscle at 28 days post-injection. At the level of the humoral response, specific VP7 antibody production was detected during eight weeks with a peak titer at four weeks post-vaccination. Other immune parameters such as respiratory burst, serum lysozyme activity, complement activity, or superoxide dismutase activity were also stimulated. Importantly, in a challenge against GCRV, the treated fish showed good protection levels even at low plasmid doses (1 µg: 73% RPS, 5 µg: 91% RPS and 10 µg: 100% RPS) when compared with the naked plasmid (1 µg: 9% RPS, 5 µg: 27% RPS and 10 µg: 44% RPS) [73] (Table 2).

### 2.3. Chitosan

Chitin is a natural, biodegradable, biocompatible, and nontoxic biopolymer derived from the shells of crustaceans, insects, and some microorganisms. It can be converted to chitosan, a linear polysaccharide compound of β-(1-4)-linked d-glucosamine and N-acetyl-d-glucosamine obtained from the *N*-deacetylated derivative of chitin by enzymatic or chemical processes. Chemical methods are used extensively for chitosan preparation for commercial purposes because of their low cost and scalability, but have a high energetic cost and produce a concentrated alkaline waste solution. In contrast, enzymatic methods offer the possibility of a controlled process, resulting in the production of well-defined chitosan [74]. Chitosan nanoparticles are prepared by ionic gelation [75], followed by freeze-drying (or spray-drying) to recover these particles.

The solubilization of chitosan occurs by protonation of the -NH_2_ group on the C-2 position of the d-glucosamine repeat unit, whereby the polysaccharide is converted to a polyelectrolyte in acidic media. Being soluble in aqueous solutions, chitosan and its derivatives are largely used in medical and pharmaceutical applications like artificial matrices for tissue engineering, targeted drug delivery, drug transport, protein delivery or gene transfer [76,77,78,79]. They can be functionalized to display antimicrobial activity against many bacteria, filamentous fungi and yeasts [80], hemostatic potential [81], and antioxidant activity [82].

The beneficial applications of chitosan on fish have been demonstrated in different studies in which chitosan nanoparticles were administrated through diet. Diets supplemented with chitosan for rainbow trout [83], olive flounder [84], koi [85], kelp grouper [86], turbot [87], gibel carp [88], mrigal carp [89], and Asian seabass [90] have proved that the chitosan could enhance growth, the innate immunity, disease, and stress resistance, improve haematological parameters and improve water quality. From fish immunological perspective, chitosan nanoparticles have been used for the delivery of vitamin C [91], RNA [92], or DNA [93,94,95] due to their positive charge and solubility in aqueous solution. In addition, chitosan protects encapsulated active compounds from the harsh conditions in the gastrointestinal tract and enhances their absorption [96]. Therefore, chitosans can be used for delivering immunostimulants or vaccines to fish in aquaculture.

#### 2.3.1. Encapsulation of Compounds in Chitosan Nanoparticles

The DNA that encodes for the 38 kDa protein of the external membrane (OMP38) of *Vibrio anguillarum* was encapsulated in chitosan and administered with food to Asian sea bass (*Lates calcarifer*). It induced a significant antibody immune response and was able to give moderate levels of protection (RPS 46%) against experimental challenge with *V. anguillarum* [94] (Table 2). Another pDNA vaccine constructed with the outer membrane protein K of *Vibrio parahaemolyticus* was encapsulated in chitosan nanoparticles and mixed with dry fish food powder and used to feed blackhead seabream (*Acanthopagrus schlegelii*). The outer membrane protein K gene and protein were expressed in muscle, liver, kidney, and mid-intestine of the vaccinated animals. Furthermore, blackhead seabream were protected from *V. parahaemolyticus* challenge with 72.3% RPS after 21 days post-vaccination [95] (Table 2). Ramos and coworkers also clearly showed that chitosan is an excellent DNA delivery system through oral administration, either by feeding with plasmid DNA-chitosan incorporated into the food, or by direct intrabuccal delivery [93] (Table 2).

In a different setup, dietary RNA (*i.e.*, nucleotides derived from yeast) was loaded into chitosan nanoparticles at a chitosan/RNA ratio of 2:1 and were fed during 60 days to fingerlings of *Labeo rohita*. The body composition in terms of protein and lipid content was not affected by RNA-loaded chitosan nanoparticles (chitosan-NPs) while the growth, performance, immunity, and survival following a bacterial challenge (*Aeromonas hydrophila*) were significantly increased compared to only chitosan or bare RNA. Unaffected glucose and serum uric acid levels, and decreased transaminases and dehydrogenases, coupled with improved performance, indicated an enhanced energetic efficiency for anabolic processes and the safety of RNA-loaded chitosan-NPs as a nutraceutical [92] (Table 2).

Finally, chitosan-NPs are very suitable to encapsulate Vitamin C. In the gastrointestinal tract of rainbow trout (*O. mykiss*), the release of Vitamin C was regulated by the chitosan encapsulation up to 48 h. The innate immunity indices (lysozyme and complement proteins) were considerably increased in the treated rainbow trout and even the non-specific defense mechanisms were stimulated as a result of the synergistic effects caused by Vitamin C and the chitosan nanoparticle itself [91] (Table 2). Vitamin C was also administered in this way to post-metamorphic larvae of *Solea senegalensis* and rotifers (*Brachionus plicatilis*). The NPs were stable in seawater and *in vitro* assays with a zebrafish liver cell-line showed a statistically significant increase in total antioxidant capacity. In addition, the nanoparticles were able to penetrate through the intestinal epithelium in *S. senegalensis* larvae and could be used as an enriching additive for rotifers [97] (Table 2).

#### 2.3.2. Encapsulation of Compounds in Chitosan Microparticles

Not only chitosan-NPs but also the larger chitosan microparticles (chitosan-MPs) are intensively studied. A plasmid containing the major capsid protein (MCP) gene of Lymphocystis Disease Virus (LCDV) was encapsulated in chitosan-MPs using an emulsion-based methodology. Oral administration led to an increase in the immune response in Japanese flounder (*Paralichythys olivaceus*) compared to injection immunization with naked plasmid DNA [52] (Table 1). The surface antigens (Ag) of the parasite *Philasterides dicentrarchi* were encapsulated and covalently linked to a polymeric microparticle formulation composed of two biodegradable polymers (chitosan and Gantrez). Poly (methyl vinyl ether)-co-(maleic anhydride) (Gantrez AN119) is a polymer belonging to the vinyl ether group that it is widely used for pharmaceutical purposes and has also been used to prepare ligand-nanoparticle conjugates for eliciting immune responses [98]. These chitosan and gantrez MPs encapsulated vaccine induced higher level of antibody than that induced by the same vaccine emulsified in FCA [53] (Table 1). These MPs could also significantly stimulate the phagocytic activity of leukocytes and the levels of the pro-inflammatory cytokine tumor necrosis factor alpha (TNFα) and also increased the production of reactive oxygen and nitrogen species in the anterior kidney of turbot (*Scophthalmus maximus*) [54] (Table 1). Finally, alginate-coated chitosan-MPs were evaluated through oral dietary administration. A potent humoral and innate immune response was elicited but it was not sufficient to induce protection against *Aeromonas hydrophila* infection under these conditions [55] (Table 1).

### 2.4. Liposomes

Liposomes are spherical, closed structures, composed of phospholipid bilayers, which enclose part of the surrounding solvent into their interior [99]. They are self-sealing and have the capacity to incorporate both hydrophilic and lipophilic drugs. Since the early 1980s, liposomes have been extensively studied as a drug carrier transport to target cells or tissues [100,101,102,103]. The drug delivery properties of liposomes are largely determined by factors such as the lipid composition, the particle size, the net charge and the loaded compound [104]. The liposome charge needs to be considered when administering molecules to fish, since fish gills contain a high level of mucin. In rainbow trout fry, a mechanism of acute toxicity after liposome treatment was suggested to be an interaction between the cationic liposomes and anionic components of gill mucin [105]. However, no toxicity was reported in zebrafish after immersion administration of nanoliposomes [23].

There is a wide variety of techniques that can be used to produce liposomal formulations, such as the Bangham method, detergent depletion method or extrusion [106]. All methods for producing liposomes require lipids to be combined by some means with an aqueous phase [107]. The extrusion technique is the most common method to prepare liposomes because it allows a better control of the size and the polydispersity index [108,109]. Extrusion is a process in which micrometric liposomes are structurally modified to large unilamellar vesicles or nanoliposomes depending on the pore-size of the filters used [108,110,111]. Compared to micro-liposomes, nanoliposomes provide more surface area and have the potential to increase solubility, enhance bioavailability, improve controlled release, and enable precision targeting of the encapsulated material to a greater extent [112].

#### 2.4.1. Encapsulation of Bacterial Antigens in Liposomes

FKB vaccines composed of liposomes entrapping *Vibrio harveyi* were tested in *Epinephelus bruneus.* In *in vivo* infection assays, the cumulative mortality was 10%, 15%, and 65% lower in this immunized group compared to treatment with *V. harveyi* alone, liposome alone and non-immunized groups, respectively [113] (Table 2). In another study in carp (*Cyprinus carpio*), the oral administration of liposomes containing *Aeromonas salmonicida* antigen was investigated*.* The survival of carp after the challenge was 83% when they were immunised with *A. salmonicida* antigen-containing liposomes, whereas non-immunized carp showed 66% survival. Furthermore, the development of skin ulcers was significantly inhibited in carp immunized with liposomes containing *A. salmonicida* antigen [114].

Lipopolysaccharide (LPS) from *A. salmonicida* was also incorporated into liposomes in order to enhance the immune response in rainbow trout (*O. mykiss*). LPS incorporated into multilamellar vesicles or large unilamellar vesicles prolonged the period of serum anti-LPS antibody levels to 6–14 weeks comparing to free-LPS (2–4 weeks) when administered intraperitoneally [115] (Table 2). Also in rainbow trout, the immune efficacy of vaccine containing liposome particles with vaccine alone against furunculosis was compared [116]. Results indicated that the protection level was significantly enhanced when the vaccine also contained liposomes (Table 2). In addition, vaccinated fish appeared to be significantly larger than control fish.

Lastly, the *A. hydrophila* antigens entrapped in liposomes were developed for oral administration to immunize common carp (*C. carpio)*. The levels of antibodies in the serum rose at two and three weeks post-vaccination and the vaccination protected the fish after injection with live *A. hydrophila* at 22 days post-vaccination [60] (Table 2).

#### 2.4.2. Encapsulation of Viral Antigens in Liposomes

Formalin-inactivated koi herpesvirus entrapped within liposomes was used for oral vaccination of common carp (*C. carpio*)*.* Specific antibody titer was significantly increased and challenge experiments revealed that orally vaccinated fish were protected from infection with two different isolates of koi herpesvirus (NKC03 and IKC03) showing high RPS (75% and 65%, respectively) [117] (Table 2).

Distinct from classical vaccines, immunostimulant-loaded liposomes are also developed to protect fish against bacterial and viral infections. For example, polyinosinic-polycytidylic acid (Poly I:C) is a synthetic analog of double-stranded RNA and is a typical molecular pattern associated with viral infections. When combined with LPS, it is a strong stimulus to the innate immune system. Liposomes encapsulating both Poly I:C and LPS elicited a pro-inflammatory and anti-viral response in zebrafish hepatocytes and trout macrophages. When administrated *in vivo* they accumulated in immune tissue and specifically in macrophages. Of interest, they protected zebrafish against otherwise lethal bacterial (*Pseudomonas aeruginosa* PAO1) and viral (Spring Viraemia of Carp Virus) infections regardless of whether they were administered by injection or by immersion. No stimulation of innate immunity was observed in the treatment with empty liposomes or with the free immunostimulants [23,118] (Table 2).

**Table 2:** Nanoparticles used as delivery system in fish.

Nanoparticle	Size	Production Technique and Composition	Encapsulated Molecule	Administration	Species	Fish Size	RPS	Reference
Calcium phosphate	224.98 ± 14.62 nm	n.d.	S-layer protein from *Aeromonas hydrophila*	i.p. injection	*Labeo rohita*	100–150 g	97% E, 13% N and 94% E with FIA at 15 DPC (*)	[119]
Carbon nanotubes	d: 10-20 nm; l: 1–2 µm	n.d., SWCNTs and MWCNTs	Sulfonate group, polyethyleglycol and sulfonic acid	*In vitro*, head kidney monocytes	*Oncorhynchus mykiss*	0.5–1 kg	n.d.	[72]
	d: 19.9 ± 8.25 nm; l: 0.8 ± 0.5 µm	n.d., MWCNTs	BSA	Microinjection	*Danio rerio*	embryos/larvae	n.d.	[71]
	n.d.	n.d., SWCNTs	Plasmid DNA: VP7 from grass carp reovirus	i.p. injection	*Ctenopharyngodon idella*	25–30 g	73% E (1 µg), 91% E (5 µg) 100% E (10 µg), 9% N (1 µg), 27% N (5 µg) and N (10 µg) at 15 DPC	[73]
Chitosan	n.d.	0.02% chitosan in sodium acetate buffer	Plasmid DNA: OMP38	Oral	*Lates calcarifer*	Juveniles	46% E at 14 DPC	[94]
	218.9 nm	TPP ionic gelation, 2 mg/mL chitosan in 3% (*v/v*) acetate	Plasmid DNA: OMPK	Oral	*Acanthopagrus schlegelii*	15–16 cm	72.3% E and 0% N 14 DPC	[95]
	n.d.	Complex coacervation, 0.02% (*w/v*) powdered chitosan	Plasmid DNA: βgalactosidase	Oral	*Oreochromis niloticus*	5–10 cm and 33–40 g	n.d.	[93]
	287.1 ± 1.49 nm	Complex coacervation, chitosan to RNA ratio: 1:1, 2:1, and 3:1	Bare RNA	Oral	*Labeo rohita*	2.7–3.1 g	83% E (2:1) and 33% N at 15 DPC (*)	[92]
	185.4 ± 2.1 nm	TPP ionic gelation, chitosan in 1% (*w/v*) acetic acid solution	Vitamin C	Oral	*Onchorhynchus mykiss*	Adult	n.d.	[91]
	253–258 nm	Ionotropic gelation, chitosan at concentration of 2.4 mg/mL in acetic acid solution (0.4% *v/v*)	Vitamin C	Oral	*Solea senegalensis*	Larvae	n.d.	[97]
Liposomes	n.d.	Film dispersion method. DPPC, DPPS, Chol (molar ratio 1:10:5)	FKB *Vibrio harveyi*	i.p.injection	*Epinephelus bruneus*	29.5 ± 2.1 g	75% E, 65% N and 60% liposome at 30 DPC	[113]
	n.d.	Film dispersion method. DPPC (0.5 µmol), DPPS (0.5 µmol) and Chol (1 µmol)	*Aeromonas salmonicida* total extract	Oral	*Cyprinus carpio*	350 g	54% E at 30 DPC (*)	[114]
	200 nm	Extrusion method. PC:Chol: PG or PC:Chol:SA in a 6:3:1 molar ratio	LPS from *Aeromonas salmonicida*	i.p.injection	*Onchorhynchus mykiss*	40 and 80 g	n.d.	[115]
	n.d.	Film dispersion method. 600 mg of phosphatidylcholine in 25 mL chloroform	FKB *Aeromonas salmonicida*, inactivated toxin and LPS	Immersion	*Salmo gairdneri*	Fry	70% E and 59% N at 126 DPC (*)	[116]
	n.d.	Film dispersion method. PS, PC, and Chol (molar ratio 1:10:5)	Koi herpesvirus whole extract	Oral	*Cyprinus carpio*	30 g	74% E (NKC03) and 65% E (IKC03) at 23 DPC	[117]
	125 nm	Extrusion method. DOPA, DLPC, Chol, Cholesteryl and Chol-PEG600	LPS and Poly I:C	*In vitro,* zebrafish hepatocytes and head kidney macrophages	*Danio rerio* and *Onchorhynchus mykiss*	Zebrafish hepatocytes, trout macrophages	n.d.	[118]
	125 nm	Extrusion method. DOPA, DLPC, Chol, Cholesteryl and Chol-PEG600	LPS and Poly I:C	Injection and immersion	*Danio rerio*	Adult	33% E, 21% N and 20% liposome at 15 DPC	[23]
	n.d.	High-pressure homogenization. 6% (*wt/v*) cinnamaldehyde, 10% (*v/v*) lecithin and 0.5% (*v/v*) α-tocopherol	Cinnamaldehyde	Immersion	*Danio rerio*	Adult	58% E at 11 DPC (*Vibrio Vulnificus*)*,* 35% E at 8 DPC (*Aeromonas hydrophila*) and 31% E at 8 DPC (*Streptococcus agalactiae*) (*)	[120]
Liposome	n.d.	Lipid film hydration, lipid:peptide ratio of 1:50	Melittin	*In vitro,* EPC cell line	*Pimephales promelas*	EPC cell line	n.d.	[121]
	n.d	Film dispersion method, DPPC (0.5 µmol), DPPS (0.5 µmol) and Chol (1 µmol), or DPPC (3.5 µmol) and Chol (1 µmol)	BSA	Oral	*Cyprinus carpio*	350 g	n.d.	[122]
PLGA	125–225 nm	D.E., PLGA: 50:50 (40–75 kDa); PLA (85–160 kDa)	OMP from *Aeromonas hydrophila*	i.p. injection	*Labaeo rohita*	50 ± 10 g	75% PLA, 55% PLGA and 38 % N at 42 DPV	[123]
	320–500 nm	D.E., n.d.	Plasmid DNA: Firefly luciferase gene	i.m. injection	*Salmo salar*	30 g	n.d.	[124]
	< 500 nm	D.E., n.d.	Plasmid DNA: MCP from LCDV	Oral	*Paralich thys olivaceus*	50–100 g	n.d.	[125]
	n.d.	D.E., 5% of PLGA/methylene chloride and 5% of PVA/water soluble	Plasmid DNA: protein-G from IHNV	Oral	*Onchorhynchus mykiss*	5 g	11% E low dose, 22% E high dose and 82% N at 180 DPC; 0% E low dose, 19% E high dose and 55% N at 300 DPC	[126]
	300–400 nm	D.E., PLGA : 50:50 (5–15 kDa; 40–75 kDa); 75:25 (66–107 kDa); PLA (24–47 kDa)	Hemocyanin from *Limulus polyphemus*	i.p. injection	*Salmo salar*	29 ± 3.1 g	n.d.	[127]
	300–400 nm	D.E., PLGA: 50:50 (5–15 kDa; 40–75 kDa); 75:25 (66–107 kDa); PLA (24–47 kDa)	β-glucan	i.p. injection	*Salmo salar*	29 ± 3.1 g	n.d.	[127]
	300–400 nm	D.E., PLGA: 50:50 (5–15 kDa; 40–75 kDa); 75:25 (66–107 kDa); PLA (24–47 kDa)	β-glucan	i.p. injection	*Salmo salar*	29 ± 3.1 g	n.d.	[127]
	< 1000 nm	D.E., n.d.	γ-globulins from human blood	i.p. injection	*Salmo salar*	30 g	n.d.	[128]
	< 1000 nm	D.E., n.d.	β-glucan	i.p. injection	*Salmo salar*	30 g	n.d.	[128]
SLN	141–335 nm	n.d.	6-Coumarin	*In vitro*, SAF-1 cell line and HK leukocytes	*Sparus aurata*	100 g	n.d.	[129]

BSA: Bovine serum albumin; Chol: Cholesterol; D.E.: double emulsion; DLPC: 1,2-didodecanoyl-sn-glycero-3-phosphocholine; DOPA: 1,2-dioleoyl-sn-glycero-3-phosphoric acid monosodium salt; DPC: days post-challenge; DPPC: Dipalmitoylphosphatidylcholine; DPPS: Dipalmitoylphosphatidylserine; E: encapsulated antigen; FIA: Freund`s incomplete adjuvant; FKB: formalin killed bacteria; i.m.: intramuscular injection; i.p.: intraperitoneal; IHNV: Infectious haematopoetic necrosis virus; LCDV: Lymphocystis disease virus; LPS: Lipopolysaccharide; MCP: major capsid protein; MWCNTs: Multi-Walled Carbon Nanotubes; N: naked antigen ; n.d.: not described; NKC03 and IKC03: two koi herpesvirus isolates; OMP: outer membrane protein; OMP38: outer membrane protein of *Vibrio anguillarum*; OMPK: Outer membrane protein K; PC: Phosphatidylcholine; PG: Phosphatidylglycerol; PLA: Polylactic acid; PLGA: poly(lactic-co-glycolic acid); Poly I:C: Polyinosinic:polycytidylic acid; PS: Phosphatidylserine; PVA: polyvinyl alcohol ; RPS: Relative percent survival; SA: Stearylamine; SWCNTs: Single-Walled Carbon Nanotubes; βgal: β-galactosidase; VP7: Viral protein 7; (*): calculated RPS.

#### 2.4.3. Encapsulation of Other Compounds in Liposomes

Cinnamaldehyde, a natural compound extracted from cinnamon, was encapsulated in liposomes. These liposomes displayed antimicrobial activity *in vitro* against aquatic pathogens such as *Streptococcus agalactiae*, *Aeromonas hydrophila*, and *Vibrio vulnificus*, as well as the antibiotic resistants *Vibrio parahaemolyticus* and *Vibrio alginolyticus*. The *in vivo* results using an immersion treatment demonstrated an increased survival rate and bacterial growth inhibition in zebrafish infected with *S. agalactiae*, *A. hydrophila* and *V. vulnificus* [120] (Table 2).

Also melittin, an antimicrobial peptide, was loaded into liposomes with covalently attached antibodies directed against Viral Haemorrhagic Septicemia Rhabdovirus (VHSV) glycoprotein G (Table 2). These melittin-immunoliposomes were capable of inhibiting the VHSV infectivity by 95% via direct inactivation of the virus. To our knowledge, this is the first report on fish pathogen targeted liposomes. However, the characterization of this formulation was not described nor the size or the charge of this formulation [121].

Finally, humoral immune responses were analyzed in a study of oral administration of liposome-entrapped BSA in carp (*C. carpio*). The BSA-containing liposomes were stable in carp bile and induced significant antibody responses against BSA in serum as well as in intestinal mucus and bile. BSA-specific antibody secreting lymphocytes were detected in the spleen and head kidney of immunized fish. In contrast, no serum antibody responses were observed when fish were orally immunized with BSA-containing unstable liposomes or BSA alone [122] (Table 2).

### 2.5. Poly (Lactic-co-Glycolic Acid) (PLGA)

PLGA, Poly (Lactic-co-Glycolic Acid) is a biodegradable polymer and probably the most extensively investigated carrier for drug delivery in mammals [130,131]. PLGA is a copolymer synthesized by two different monomers, lactic acid and glycolic acid. The forms of PLGA depend on the monomer ratio used during the polymerization process. PLGA nanoparticles (PLGA-NPs) are degraded by hydrolysis and the degradation time depends on the monomer ratio and on the molecular weight of the polymers [132]. The PLGA-NPs were approved for human use by the Food and Drug Administration (FDA, USA) and the European Medicines Agency (EMA), because they are highly biodegradable and biocompatible [133]. When the polymer is hydrolyzed the glycolic and lactic acid monomers are released and are eventually removed from the body through the citric acid cycle [134]. For this reason, there is minimal toxicity associated with the use of PLGA as a nanodelivery system since these components are present in different metabolic pathways. The most common PLGA-NPs preparation method used in fish is the double emulsion method [123,124,125,126,127,128] which is based on the dissolution of an appropriate amount of polymer (PLGA) in an organic solvent (oil phase) such as dichloromethane, chloroform or ethylacetate [135]. Hydrophobic drugs can be added directly to the oil phase, whereas hydrophilic drugs must be first emulsified with the polymer solution prior to the formation of particles [136]. Then, the solution is emulsified by the addition of an aqueous solution containing a surfactant or an emulsifying agent (e.g., polyvinyl alcohol). By reducing the pressure or by continuous stirring the organic solvent evaporates and this results in the formation of solid nanoparticles. The encapsulation efficiency and the particle size can be controlled by the solvent choice and the stirring rate [135].

The internalization of PLGA-NPs by cells involves different uptake mechanisms. In mammals, the PLGA-NPs are able to avoid the endo-lysosomal system and are maintained in the cytoplasm. The uptake mechanisms of PLGA-NPs in teleosts is poorly understood and few published work have addressed this issue [118]. Despite this, it is known that nanoparticles of less than 500 nm in size are able to enter the bloodstream and subsequently they are cleared by phagocytes in the head kidney, spleen, and/or liver [137]. PLGA-NPs are highly versatile loading bioactive compounds. For applications in fish, γ-globulins from human blood, β-glucan [127,128], DNA vaccines [124,125,126] or the bacterial outer membrane complex [123] have been encapsulated (Table 2).

#### 2.5.1. Encapsulation of Bacterial Antigens in PLGA-NPs and -MPs

PLGA-NPs were compared with polylactic acid-NPs (PLA-NPs), which also have good mechanical strength, to encapsulate the outer membrane complex from *A. hydrophyla* [138,139]. This complex consists mainly of lipopolysaccharide, phospholipids, and a group of outer membrane proteins. The encapsulation efficiency was higher in PLGA-NPs compared with PLA-NPs (59% and 44%, respectively) but the release *in vitro* was slower from PLA-NPs than from PLGA-NPs (50% at 24 h and 4 h, respectively). This might be explained by the higher hydrophilic nature of the PLGA- compared to PLA-NPs. The non-specific and specific immunity were stimulated in *L. rohita* by both PLGA- and PLA-NPs and this at higher levels than the naked antigen (Table 2). Finally, in a challenge against *A. hydrophyla*, the PLA-vaccine provided higher levels of protection compared with the PLGA-vaccine (75% RPS and 55% RPS, respectively) and with the naked antigen (38% RPS) [123]. In addition, the encapsulation of the same antigen in PLGA-microparticles (PLGA-MPs) was evaluated [56]. These PLGA-MPs were studied in *L. rohita* administrated parenterally (Table 2). Encapsulation efficiency of PLGA-MPs was lower compared to PLGA-NPs (25% and 50%, respectively). Both the microparticles and the nanoparticles significantly stimulated non-specific (myeloperoxidase, respiratory burst activity, haemagglutination, *etc.*) and specific immune response parameters at similar levels at 21 and 42 days after vaccination. Finally, in a challenge study PLGA-NPs provided protection (55% RPS) against *A. hydrophila* infection, while no data was reported for PLGA-MPs [123].

Oligodeoxynucleotides (ODNs) are short single-stranded synthetic DNA molecules that contain unmethylated CpG motifs. These motifs are highly abundant in bacterial DNA and extremely rare in vertebrates, and they are classified as a type of *Pathogen Associated Molecular Pattern* (PAMP). In mammals, they are recognized by Toll-like receptor 9 leading to strong immunostimulatory effects and also fish are able respond to CpG binding to TLR9 [140]. This antigen was encapsulated in PLGA/liposome-MPs and used to stimulate the immune system of *E. bruneus* using intraperitoneal injection [61] (Table 2). Superoxide dismutase, respiratory burst, and complement activity were mainly stimulated by the PLGA/Liposome microparticles. In contrast, the adaptive immune response and the specific *V. alginolyticus* serum antibody levels were significantly higher with the PLGA-MPs. Finally, the treatment with the PLGA-, Liposome- and PLGA/Liposome-MPs encapsulating ODNs provided good protection levels (78%, 83%, and 83% of RPS, respectively) against a *V. alginolyticus* infection [61] but they are not significantly different from the naked ODN (78% RPS).

The alginate-MPs mentioned in section 2.1.1 were compared with PLGA-MPs as vehicles for the delivery of FKB from *L. garvieae* (Table 2). The PLGA-NPs provided similar protection levels than alginate-FKB-LG (about 63% RPS at 30 days), but lower when compared with the conventional FKB vaccine intraperitoneally injected (95% RPS) [45].

Hølvold *et al.* [124] encapsulated in PLGA-NPs a plasmid containing the firefly luciferase gene under the control of the CMV-IEP promoter. Despite the fact that this formulation does not contain a specific bacterial antigen, the plasmid itself is from bacterial origin and acts as an immunostimulant (bacterial CpG). The PLGA-NPs showed a fast release of the plasmid (80% after 1 h), induced a significant increase in IL-1β and IFNα gene expression in muscle at the injection site in comparison with naked plasmid and stimulated TNFα expression in head kidney. The PLGA-NPs labeled with [^125^I]-fluorescein were detected until day 70 in trunk kidney, muscle and organ package (liver, heart, gastrointestinal tract and interstitial adipose tissue) [124]. The performance of these NPs was here also compared to MPs. The PLGA-MPs showed a lower release of antigen than the PLGA-NPs (49% at 1 h and an accumulative release of 69% at day 70; 81% at 1 h and an accumulative release of 96% at day 70, respectively). Additionally, PLGA-MPs had a higher retention than PLGA-NPs at the injection site, contributing to the onset of severe histopathological inflammation. This suggests that nanoparticles are more suited to avoid potential tissue damage. Both PLGA-MPs and PLGA-NPs showed better performance than naked plasmid DNA for the induction of pro-inflammatory and antiviral immune responses [124].

#### 2.5.2. Encapsulation of Viral Antigens in PLGA-NPs and -MPs

PLGA-NPs have been mainly used to encapsulate DNA vaccines aiming to protect against viral diseases. Lymphocystis Disease Virus (LCDV) infection is not lethal, but infected fish are more susceptible to secondary microbial infection [141]. The progression of the disease correlates with an increase in the presence of nodules. A plasmid coding for the major capsid protein (MCP) of LCDV was encapsulated in PLGA-NPs and PLGA-MPs and tested in *P. olivaceus* [125]. The encapsulation efficiency was 64% and full release (100%) was achieved after 60 h at pH 2.0 and after 90 h at pH 9.0. MCP gene expression was detected in gills, intestine, spleen, and kidney from 10 to 90 days after oral administration. Specific serum antibody titers against LCDV reached a maximum at 30 days post-administration. Importantly, in a challenge against LCDV, the presence of nodules was significantly lower in PLGA-vaccinated fish compared to naked DNA vaccinated fish (17% *versus* 100%, respectively) [125]. Of note, Tian and coworkers showed that the encapsulation efficiency in PLGA microparticles was more stable than in the nanoparticle system (78%–88% and 64%–96%, respectively) [57,125]. PLGA-NPs and PLGA-MPs displayed similar performance except that the nanoparticles showed higher release characteristics. The study concluded that PLGA-MPs were also effective oral carriers for the transfer of plasmid DNA [57].

Other PLGA-NPs containing a DNA vaccine against Infectious Haematopoetic Necrosis Virus (IHNV) were used to vaccinate *O. mykiss* (Table 2). In this case, the release of plasmid DNA was not clearly pH dependent, nor were there significant differences between the number of fish expressing the plasmid gene compared to the naked plasmid treatment. This PLGA-vaccine was also not able to confer protection against IHNV [126].

#### 2.5.3. Encapsulation of Other Antigens into PLGA-NPs and -MPs

As mentioned above, PLGA-NPs allow maximal versatility in encapsulating molecules of different nature. Other representative examples of this are immunostimulants such as γ-globulins from human blood, β-glucan from *L. hyperborea* and hemocyanin from *Limulus polyphemus* (Table 2). Three different loaded PLGA-NPs (β-glucan, hemocyanin and both combined) were administrated by intraperitoneal injection in *S. salar* (Table 2). The gene expression profile showed that even PLGA-NPs alone induced a mild inflammatory response in *S. salar* having potential as an adjuvant in salmon vaccine [127]. In a subsequent study, the same group assessed different formulations of PLGA-NPs and -MPs at different monomer ratios. The release of the antigen was similar (around 10%) for all formulations, however the nanoparticles co-encapsulating γ-globulins and β-glucan induced the highest specific antibody response [128]. In another study, PLGA-MPs loaded with γ-globulins were also investigated in *S. salar* by oral administration. The encapsulation in PLGA-MPs allowed its stability in the stomach for longer periods of time, slowing down the passage into the intestine and increasing the levels of intact antigen reaching the blood stream. Also, the PLGA-MPs stimulated the antibody titer in serum but not in cutaneous mucus, gut mucus, or in bile [58].

Parasitic protozoa have developed sophisticated evasion mechanisms to evade the host’s innate immune defenses and currently, there are no anti-parasitic vaccines commercially available for farmed fish. A unique study aimed to design a specific delivery system for parasite disease prevention [59]. Formalin-killed parasite (i-antigen) from *Uronema marimun,* an opportunistic pathogen infecting flounder (*P. olivaceus*) and grouper (*E. bruneus*), was encapsulated into PLGA-MPs. The PLGA-MPs were administrated to *E. bruneus* by intraperitoneal injection and different innate immune response parameters such as respiratory burst activity, serum lysozyme activity, or complement activity were evaluated. All of them were stimulated by PLGA-MPs and were sustained from one to four weeks, whereas the treatment with the free i-antigen was detected only at week four and at lower levels. The specific i-antigen antibody levels were stimulated both by the free i-antigen and the PLGA-i-antigen, but again at higher levels by the PLGA-MPs [59]. Interestingly, the levels of protection from the loaded PLGA-MPs against *U. marinum* infection were notably high (only 20% of cumulative mortality after 30 days), but only slightly different from the empty PLGA-MPs or the free i-antigen [59].

### 2.6. Other Nanodelivery Systems

Although the amount of research done in mammals and fish is not comparable, there exists a large effort to discover new nanodelivery systems in teleost to cover the different needs for the prevention of diseases in aquaculture. Here we mention two additional approaches aiming to develop new nanomaterials for *in vivo* delivery in fish: calcium phosphate nanoparticles (CaP-NPs) [119] and solid lipid nanoparticles (SL-NPs) [129].

Calcium phosphate is a natural, inorganic, and biocompatible material. CaP-NPs are synthesized using different methods such as mechanochemical synthesis, combustion preparation, wet chemistry techniques, and others [142]. CaP-NPs can be produced in different morphologies, such as spheres, plate-like crystals, needles, or blades [142,143], however, the size and the stability of CaP-NPs are very difficult to control [143]. CaP-NPs are a potential nanodelivery system due to their high bioactivity, biocompatibility, biodegradability and strong adsorption ability under physiological conditions. In mammals, they have been used as nanodelivery system for drugs, vectors, antibacterial agents, or as a vaccine adjuvant [142]. In *L. rohita* CaP-NPs loaded with the S-layer from *A. hydrophila* was assessed by intraperitonal injection (Table 2). The non-specific immune responses (superoxide dismutase, myeloperoxidase, respiratory burst, *etc.*) and the specific immune response (antibody titers) were stimulated and detected at 21, 42, and 63 days post-vaccination. When fish were challenged with *A. hydrophila*, loaded CaP-NPs were able to provide good levels of protection 15 days post-vaccination (97% RPS) with a significant difference in comparison with non-encapsulated S-layer (13% RPS), but with similar level (94% of RPS) when compared with CaP-NPs with only Freund`s incomplete adjuvant [119].

Solid lipid nanoparticles are produced in solution using solid lipidic materials with surfactants that confer stability and co-surfactants that confer specific ligand properties [144]. SL-NPs can be prepared by different techniques, such as high-pressure homogenization, high-shear mixing, ultrasound, or solvent emulsification/evaporation methods [143,145]. Additionally, SL-NPs present a range of characteristic advantages, such as biocompatibility, non-toxicity, high bioavailability, high-antigen loading ability, controlled release, physical stability, and protection of encapsulated antigens. Finally, SL-NPs can be easily scaled-up for industrial purposes [145,146]. A preliminary *in vitro* characterization of SL-NPs was performed in fish [129] (Table 2). The loaded SL-NPs had a mean diameter of 235–335 nm depending on the amount of cargo, with a net surface charge between −12.5 and −16.5 mV. These nanoparticles were loaded with a fluorescent molecule (6-Coumarin) and were tested for uptake and toxicity in a cell line (SAF-1) and in leukocyte primary cell culture of *S. aurata* head kidney. Release of 6-Coumarin from SL-NPs was around 1% over the course of 48 h at 22 °C and both the cell line and the primary leukocytes were able to internalize these SL-NPs without affecting the cell viability. SL-NPs internalization was dose- and time-dependent. The uptake in SAF-1 cells decreased over time indicating that the SL-NPs in SAF-1 cells are likely processed in the endolysosomal compartment, while the fluorescent signal was stable over the time in primary leukocytes [129].

## 3. Discussion

Nowadays there is a large variety of materials that can be used as delivery systems for vaccine/immunostimulant administration in fish. This diversity provides a wide range of options to respond to the high number of different farmed species and the challenge to achieve a good health status in the presence of different potentially harmful microorganisms. However, the study of nanoparticles for aquacultural use is still in its early stage. Research shows variable efficiency of protection depending on the nature of the nanomaterials, the method to produce the nanoparticles, the antigens encapsulated or the fish species assessed. Often there is a lack of information about the manufacturing process as well as the physico-chemical characteristics of the nanoparticles and the properties of the antigens after the encapsulation process, hampering a correct analysis and comparison between delivery systems. For instance, not all publications mention the size of the particles, the efficiency of encapsulation or the release of the antigens in *in vitro* conditions (see Table 1 and Table 2). In many cases, the starting point is the “recycling” of particles for mammalian use that are applied directly in fish, without considering the evident differences between mammals and fish. The researcher working on fish health should make a strong effort to design or adapt nanoparticles in order to reach optimal compatibility with the fish characteristics. However, this is difficult because the fish immune system has several differences with the mammalian immune system regarding cell types, cell biology, tissues involved in immune response, *etc.* Also, the fish immune system is not well known in many aspects such as how the adaptive system memory works, which cell types are involved or the role of mucosal immunity.

In general, the use of microparticles is more frequent than the use of nanoparticles even when the surface area/volume ratio is much more advantageous in nanoparticles. An explanation of this is that some materials are not easy to manipulate in the nanosize range or there are not protocols to nanosize such materials. An example is alginate that has been mainly used to produce microparticles and only recently has it started to be used to produce sub-200 nm particles [147]. Again, the characterization of the manufactured nanoparticles should be detailed in the publications in order to compare between the administration routes, the adjuvant properties, the potential degradation of the loaded compound, the efficacy of the system protecting against infection, and the targeted cells or tissues. Different fish species have different responses to vaccination [148] and this fact should be considered when choosing an encapsulation system because they may not be transferable from one species to another. Additionally, the encapsulated antigens modify the physico-chemical characteristics of the nanodelivery system so that the results of the assays on stability, size, surface charge, and organ biodistribution cannot be extrapolated from one molecule to another using the same encapsulating particle. Similarly, the characteristics of the antigen can be changed when it is encapsulated, and thus the functional structure, stability, and immunogenicity of the antigen need to be verified. For example, the size and the surface charge are extremely important for interaction with cells and should be characterized in the loaded system because they can change easily [118]. Of note, in some cases the encapsulation did not provide any protection [40] or did not improve the protection with respect to conventional immunoprotective therapies [55]. Overall, the administration of nanoparticles by intraperitoneal injection achieves good protection levels against infections while the oral administration is at this moment less efficient. One of the exceptions is the system developed with alginate or chitosan to encapsulate DNA vaccines [41,42]. DNA vaccines are still under development and only one commercial vaccine has been licensed in Canada. They are the most promising tools to fight viral infections and thus, the development of novel encapsulation systems to improve their administration and the efficiency is very important. Several new nanomaterials such as carbon nanotubes or solid lipid NP are still in the early steps of development but have shown promising results. For example, CNTs have been very effective for encapsulating a DNA vaccine and to confer protection against infection even at low DNA concentrations [73]. It is important to mention that in some studies, the adjuvant effect of the nanodelivery system is almost as potent as the loaded antigen itself. The adjuvant effect of the system itself has been extensively reported in mammals (e.g., liposomes) and it is also clearly observed in some fish species [124] but not in others [23].

Also an important point that should be taken into consideration is the final cost of the encapsulation system for industrial production. Some of the systems developed under research conditions are expensive and may not be affordable for the fish farmers. Finally, all of the nanodelivery systems included in this review have been characterized as non-toxic for cells (*in vitro* viability assays) and similarly *in vivo*. However, the toxicity of nanomaterials and, more importantly, the toxicity of the nanomaterial degradation products that could be detected in water should be addressed carefully [149,150].

## 4. Conclusions

Altogether, nano-encapsulation is a very promising strategy with a potential to substantially improve the development of effective vaccines for farmed fish. The research on the delivery of viral vaccines using nanoparticles will be the more important milestone in fish vaccinology. In this context, more traditional biomaterials such as alginate and chitosan have shown good results but new materials such as CNTs or solid lipid NP could improve the delivery of DNA vaccines. More research is still needed to specifically design encapsulation systems adapted to the fish immune system and to decipher the basis of the fish immune system.

## References

[1] Duff D. (1942). The oral immunization of trout against *Bacterium salmonicida*. J. Immunol..

[2] Hastein T., Gudding R., Evensen O. (2005). Bacterial vaccines for fish—An update of the current situation worldwide. Dev. Biol..

[3] Sommerset I., Krossoy B.B.E., Frost P. (2005). Vaccines for fish in aquaculture. Expert Rev. Vaccines.

[4] Mitchell H. (1995). Choosing a furunculosis vaccine: Points to consider. Bull. Aquac. Assoc. Can..

[5] Kibenge F.S.B., Godoy M.G., Fast M., Workenhe S., Kibenge M.J.T. (2012). Countermeasures against viral diseases of farmed fish. Antivir. Res..

[6] Koppang E.O., Haugarvoll E., Hordvik I., Aune L., Poppe T.T. (2005). Vaccine-associated granulomatous inflammation and melanin accumulation in Atlantic salmon, *Salmo salar* L., white muscle. J. Fish Dis..

[7] Midtlyng P.J. (1996). A field study on intraperitoneal vaccination of Atlantic salmon (*Salmo salar* L) against furunculosis. Fish Shellfish Immunol..

[8] Anderson D.P. (1992). Immunostimulants, adjuvants, and vaccine carriers in fish: Applications to aquaculture. Ann. Rev. Fish Dis..

[9] Lund R.A., Midtlyng P.J., Hansen L.P. (1997). Post-vaccination intra-abdominal adhesions as a marker to identify Atlantic salmon, *Salmo salar* L., escaped from commercial fish farms. Aquaculture.

[10] Sorum U., Damsgard B. (2004). Effects of anaesthetisation and vaccination on feed intake and growth in Atlantic salmon (*Salmo salar* L.). Aquaculture.

[11] Berg A., Rødseth O.M., Tangerås A., Hansen T. (2006). Time of vaccination influences development of adhesions, growth and spinal deformities in Atlantic salmon *Salmo salar*. Dis. Aquat. Organ.

[12] Horne M.T. (1996). Technical aspects of the administration of vaccines. Dev. Boil. Stand..

[13] Nakanishi T., Ototake M. (1996). Antigen uptake and immune responses after immersion vaccination. Dev. Boil. Stand..

[14] Kai Y.H., Chi S.C. (2008). Efficacies of inactivated vaccines against betanodavirus in grouper larvae (*Epinephelus coioides*) by bath immunization. Vaccine.

[15] Quentel C., Vigneulle M. (1996). Antigen uptake and immune responses after oral vaccination. Dev. Boil. Stand..

[16] Plant K.P., LaPatra S.E. (2011). Advances in fish vaccine delivery. Dev. Comp. Immunol..

[17] Shi J., Votruba A.R., Farokhzad O.C., Langer R. (2010). Nanotechnology in drug delivery and tissue engineering: From discovery to applications. Nano Lett..

[18] Yatvin M.B., Kreutz W., Horwitz B.A., Shinitzky M. (1980). pH-sensitive liposomes: possible clinical implications. Science.

[19] Toyokawa H., Nakao A., Bailey R.J., Nalesnik M.A., Kaizu T., Lemoine J.L., Ikeda A., Tomiyama K., Papworth G.D., Huang L. (2008). Relative contribution of direct and indirect allorecognition in developing tolerance after liver transplantation. Liver Transplantat..

[20] Li S.D., Huang L. (2008). Pharmacokinetics and biodistribution of nanoparticles. Mol. Pharm..

[21] Hillaireau H., Couvreur P. (2009). Nanocarriers’ entry into the cell: Relevance to drug delivery. Cell. Mol. Life Sci..

[22] Canton I., Battaglia G. (2012). Endocytosis at the nanoscale. Chem. Soc. Rev..

[23] Ruyra A., Cano-Sarabia M., Garcia-Valtanen P., Yero D., Gibert I., Mackenzie S.A., Estepa A., Maspoch D., Roher N. (2014). Targeting and stimulation of the zebrafish (*Danio rerio*) innate immune system with LPS/dsRNA-loaded nanoliposomes. Vaccine.

[24] Handy R.D., Von der Kammer F., Lead J.R., Hassellov M., Owen R., Crane M. (2008). The ecotoxicology and chemistry of manufactured nanoparticles. Ecotoxicology.

[25] Doshi N., Mitragotri S. (2009). Designer biomaterials for nanomedicine. Adv. Funct. Mater..

[26] Gratton S.E., Ropp P.A., Pohlhaus P.D., Luft J.C., Madden V.J., Napier M.E., DeSimone J.M. (2008). The effect of particle design on cellular internalization pathways. Proc. Natl. Acad. Sci..

[27] Champion J.A., Mitragotri S. (2006). Role of target geometry in phagocytosis. Proc. Natl. Acad. Sci..

[28] Devine D.V., Wong K., Serrano K., Chonn A., Cullis P.R. (1994). Liposome-complement interactions in rat serum—Implications for liposome survival studies. BBA Biomembr..

[29] Verma A., Stellacci F. (2010). Effect of surface properties on nanoparticle-cell interactions. Small.

[30] Cho E.C., Xie J.W., Wurm P.A., Xia Y.N. (2009). Understanding the role of surface charges in cellular adsorption *versus* internalization by selectively removing gold nanoparticles on the cell surface with a I-2/KI etchant. Nano Lett..

[31] Miller C.R., Bondurant B., McLean S.D., McGovern K.A., O’Brien D.F. (1998). Liposome-cell interactions *in vitro*: Effect of liposome surface charge on the binding and endocytosis of conventional and sterically stabilized liposomes. Biochemistry.

[32] Mislick K.A., Baldeschwieler J.D. (1996). Evidence for the role of proteoglycans in cation-mediated gene transfer. Proc. Natl. Acad. Sci..

[33] Mohanraj V.J., Chen Y. (2007). Nanoparticles—A review. Trop. J. Pharm. Res..

[34] Gombotz W.R., Wee S.F. (2012). Protein release from alginate matrices. Adv. Drug Deliv. Rev..

[35] Østgaard K., Knutsen S.H., Dyrset N., Aasen I.M. (1993). Production and characterization of guluronate lyase from *Klebsiella pneumoniae* for applications in seaweed biotechnology. Enzyme Microbial. Technol..

[36] De Vos P., Lazarjani H.A., Poncelet D., Faas M.M. (2014). Advanced drug delivery reviews. Adv. Drug Deliv. Rev..

[37] Sosnik A. (2014). Alginate particles as platform for drug delivery by the oral route: State-of-the-Art. ISRN Pharm..

[38] Tanaka H., Matsumura M. (1984). Diffusion characteristics of substrates in Ca-alginate gel beads. Biotechnol. Bioeng..

[39] Rodrigues A.P., Hirsch D., Figueiredo H.C.P., Logato P.V.R., Moraes Â.M. (2006). Production and characterisation of alginate microparticles incorporating Aeromonas hydrophila designed for fish oral vaccination. Proc. Biochem..

[40] Leal C.A.G., Carvalho-Castro G.A., Sacchetin P.S.C., Lopes C.O., Moraes Â.M., Figueiredo H.C.P. (2010). Oral and parenteral vaccines against *Flavobacterium columnare*: evaluation of humoral immune response by ELISA and *in vivo* efficiency in Nile tilapia (*Oreochromis niloticus*). Aquacult. Int..

[41] Tian J., Sun X., Chen X. (2008). Formation and oral administration of alginate microspheres loaded with pDNA coding for lymphocystis disease virus (LCDV) to Japanese flounder. Fish Shellfish Immunol..

[42] Ana I., Saint-Jean S.R., Pérez-Prieto S.I. (2010). Immunogenic and protective effects of an oral DNA vaccine against infectious pancreatic necrosis virus in fish. Fish Shellfish Immunol..

[43] Pinto Reis C., Neufeld R.J., Ribeiro A.J., Veiga F. (2006). Nanoencapsulation I. Methods for preparation of drug-loaded polymeric nanoparticles. Nanomedicine.

[44] Romalde J.L., Luzardo-Alvárez A., Ravelo C., Toranzo A.E., Blanco-Méndez J. (2004). Oral immunization using alginate microparticles as a useful strategy for booster vaccination against fish lactoccocosis. Aquaculture.

[45] Altun S., Kubilay S., Ekici S., Didinen B., Diler O. (2010). Oral vaccination against lactococcosis in rainbow trout (*Oncorhynchus mykiss*) using sodium alginate and poly (lactide-co-glycolide) carrier. Kafkas Univ. Vet. Fak. Derg..

[46] Sarmento B., Ferreira D., Veiga F., Ribeiro A. (2006). Characterization of insulin-loaded alginate nanoparticles produced by ionotropic pre-gelation through DSC and FTIR studies. Carbohydr. Polym..

[47] Sarmento B., Ribeiro A.J., Veiga F., Ferreira D.C., Neufeld R.J. (2007). Insulin-loaded nanoparticles are prepared by alginate ionotropic pre-gelation followed by chitosan polyelectrolyte complexation. J. Nanosci. Nanotech..

[48] Ahmad Z., Khuller G.K. (2008). Alginate-based sustained release drug delivery systems for tuberculosis. Expert Opin. Drug Deliv..

[49] Fonte P., Araújo F., Silva C., Pereira C., Reis S., Santos H.A., Sarmento B. (2015). Fonte. Polymer-based nanoparticles for oral insulin delivery: Revisited approaches. Biotechnol. Adv..

[50] Grabowski L.D., LaPatra S.E., Cain K.D. (2004). Systemic and mucosal antibody response in tilapia, *Oreochromis niloticus* (L.), following immunization with *Flavobacterium columnare*. J. Fish Dis..

[51] Dhar A.K., Manna S.K., Thomas Allnutt F.C. (2014). Viral vaccines for farmed finfish. Virus Dis..

[52] Tian J., Yu J., Sun X. (2008). Chitosan microspheres as candidate plasmid vaccine carrier for oral immunisation of Japanese flounder (*Paralichthys olivaceus*). Vet. Immunol. Immunopathol..

[53] León-Rodríguez L., Luzardo-Álvarez A., Blanco-Méndez J., Lamas J., Leiro J. (2012). A vaccine based on biodegradable microspheres induces protective immunity against scuticociliatosis without producing side effects in turbot. Fish Shellfish Immunol..

[54] León-Rodríguez L., Luzardo-Álvarez A., Blanco-Méndez J., Lamas J., Leiro J. (2013). Biodegradable microparticles covalently linked to surface antigens of the scuticociliate parasite *P. dicentrarchi* promote innate immune responses *in vitro*. Fish Shellfish Immunol..

[55] Behera T., Swain P. (2014). Antigen encapsulated alginate-coated chitosan microspheres stimulate both innate and adaptive immune responses in fish through oral immunization. Aquacult. Int..

[56] Behera T., Nanda P.K., Mohanty C., Mohapatra D., Swain P., Das B.K., Routray P., Mishra B.K., Sahoo S.K. (2010). Parenteral immunization of fish, *Labeo rohita* with Poly d,l-lactide-co-glycolic acid (PLGA) encapsulated antigen microparticles promotes innate and adaptive immune responses. Fish Shellfish Immunol..

[57] Tian J., Sun X., Chen X., Yu J., Qu L., Wang L. (2008). The formulation and immunisation of oral poly (dl-lactide-co-glycolide) microcapsules containing a plasmid vaccine against lymphocystis disease virus in Japanese flounder (*Paralichthys olivaceus*). Int. Immunopharmacol..

[58] Lavelle E.C., Jenkins P.G., Harris J.E. (1997). Oral immunization of rainbow trout with antigen microencapsulated in poly (dl-lactide-co-glycolide) microparticles. Vaccine.

[59] Harikrishnan R., Balasundaram C., Heo M.S. (2012). Poly d,l-lactide-co-glycolic acid (PLGA)-encapsulated vaccine on immune system in *Epinephelus bruneus* against *Uronema marinum*. Exp. Parasitol..

[60] Yasumoto S., Yoshimura T., Miyazaki T. (2006). Oral immunization of common carp with a liposome vaccine containing *Aeromonas hydrophila* antigen. Fish Pathol..

[61] Harikrishnan R., Balasundaram C., Heo M.-S. (2012). Poly d,l-lactide-co-glycolic acid-liposome encapsulated ODN on innate immunity in *Epinephelus bruneus* against *Vibrio alginolyticus*. Vet. Immunol. Immunopathol..

[62] Iijima S. (1991). Helical microtubules of graphitic carbon. Nature.

[63] Pagona G., Tagmatrachis N. (2006). Carbon nanotubes: Materials for medicinal chemistry and biotechnological applications. Curr. Med. Chem..

[64] He H., Pham-Huy L.A., Dramou P., Xiao D., Zuo P., Pham-Huy C. (2013). Carbon nanotubes: Applications in pharmacy and medicine. Bio. Med. Res. Int..

[65] Dumortier H., Lacotte S., Pastorin G., Marega R., Wu W., Bonifazi D., Briand J.P., Prato M., Muller S., Bianco A. (2006). Functionalized carbon nanotubes are non-cytotoxic and preserve the functionality of primary immune cells. Nano Lett..

[66] Kostarelos K., Bianco A., Prato M. (2009). Promises, facts and challenges for carbon nanotubes in imaging and therapeutics. Nature Nanotechnol..

[67] Pantarotto D., Partidos C.D., Hoebeke J., Brown F., Kramer E., Briand J.P., Muller S., Prato M., Bianco A. (2003). Immunization with peptide-functionalized carbon nanotubes enhances virus—Specific neutralizing antibody responses. Chem. Biol..

[68] Pantarotto D., Singh R., McCarthy D., Erhardt M., Briand J.P., Prato M., Kostarelos K., Bianco A. (2004). Functionalized carbon nanotubes for plasmid DNA gene delivery. Angew. Chem..

[69] Chakravarty P., Marches R. (2008). Thermal ablation of tumor cells with antibody-functionalized single-walled carbon nanotubes. Proc. Natl. Acad. Sci..

[70] Yacoby I., Benhar I. (2008). Antibacterial nanomedicine. Nanomedicine.

[71] Cheng J., Chan C.M., Veca L.M., Poon W.L., Chan P.K., Qu L., Sun Y.P., Cheng S.H. (2009). Acute and long-term effects after single loading of functionalized multi-walled carbon nanotubes into zebrafish (*Danio rerio*). Toxicol. Appl. Pharmacol..

[72] Klaper R., Arndt D., Setyowati K., Chen J., Goetz F. (2010). Functionalization impacts the effects of carbon nanotubes on the immune system of rainbow trout, *Oncorhynchus mykiss*. Aqua. Toxicol..

[73] Zhu B., Liu G.L., Gong Y.X., Ling F., Wang G.X. (2015). Protective immunity of grass carp immunized with DNA vaccine encoding the vp7 gene of grass carp reovirus using carbon nanotubes as a carrier molecule. Fish Shellfish Immunol..

[74] Younes I., Rinaudo M. (2015). Chitin and chitosan preparation from marine sources. Structure, properties and applications. Mar. Drugs.

[75] Calvo P., Remunan-Lopez C., Vila-Jato J.L., Alonso M.J. (1997). Development of positively charged colloidal drug carriers: Chitosan-coated polyester nanocapsules and submicron-emulsions. Colloid. Polym. Sci..

[76] Pan Y., Li Y.J., Zhao H.Y., Zheng J.M., Xu H., Wei G., Hao J.S. (2002). Bioadhesive polysaccharide in protein delivery system: chitosan nanoparticles improve the intestinal absorption of insulin *in vivo*. Int. J. Pharm..

[77] Lavertu M., Methot S., Tran-Khanh N., Buschmann M.D. (2006). High efficiency gene transfer using chitosan/DNA nanoparticles with specific combinations of molecular weight and degree of deacetylation. Biomaterials.

[78] Wei H., Zhang X., Cheng C., Cheng S.X., Zhuo R.X. (2007). Self-assembled, thermosensitive micelles of a star block copolymer based on PMMA and PNIPAAm for controlled drug delivery. Biomaterials.

[79] Kim I.Y., Seo S.J., Moon H.S., Yoo M.K., Park I.Y., Kim B.C., Cho C.S. (2008). Chitosan and its derivatives for tissue engineering applications. Biotechnol. Adv..

[80] Kong M., Chen X.G., Xing K., Park H.J. (2010). Antimicrobial properties of chitosan and mode of action: A state of the art review. Int. J. Food Microbial..

[81] Rao S.B., Sharma C.P. (1997). Use of chitosan as a biomaterial: studies on its safety and hemostatic potential. J. Biomed. Mat. Res..

[82] Chiang M.T., Yao H.T., Chen H.C. (2000). Effect of dietary chitosans with different viscosity on plasma lipids and lipid peroxidation in rats fed on a diet enriched with cholesterol. Biosci. Biotechnol. Biochem..

[83] Meshkini S., Tafy A.A., Tukmechi A., Farhang-Pajuh F. (2012). Effects of chitosan on hematological parameters and stress resistance in rainbow trout (*Oncorhynchus mykiss*). Vet. Res. Forum.

[84] Cha S.H., Lee J.S., Song C.B., Lee K.J., Jeon Y.J. (2008). Effects of chitosan-coated diet on improving water quality and innate immunity in the olive flounder, *Paralichthys olivaceus*. Aquaculture.

[85] Lin S., Pan Y., Luo L., Luo L. (2011). Effects of dietary beta-1, 3-glucan, chitosan or raffinose on the growth, innate immunity and resistance of koi (*Cyprinus carpio koi*). Fish Shellfish Immunol..

[86] Harikrishnan R., Kim J.S., Balasundaram C., Heo M.S. (2012). Dietary supplementation with chitin and chitosan on haematology and innate immune response in *Epinephelus bruneus* against *Philasterides dicentrarchi*. Exp. Parasitol..

[87] Cui L.Q., Xu W., Ai Q.H., Wang D.F., Mai K.S. (2013). Effects of dietary chitosan oligosaccharide complex with rare earth on growth performance and innate immune response of turbot, *Scophthalmus maximus* L.. Aquac. Res..

[88] Chen Y., Zhu X., Yang Y., Han D., Jin J., Xie S. (2014). Effect of dietary chitosan on growth performance, haematology, immune response, intestine morphology, intestine microbiota and disease resistance in gibel carp (*Carassius auratus gibelio*). Aquacult. Nutr..

[89] Mari L.S.S., Jagruthi C., Anbazahan S.M., Yogeshwari G., Thirumurugan R., Arockiaraj J., Mariappan P., Balasundaram C., Harikrishnan R. (2014). Protective effect of chitin and chitosan enriched diets on immunity and disease resistance in *Cirrhina mrigala* against *Aphanomyces invadans*. Fish Shellfish Immunol..

[90] Ranjan R., Prasad K.P., Vani T., Kumar R. (2014). Effect of dietary chitosan on haematology, innate immunity and disease resistance of Asian seabass *Lates calcarifer* (Bloch). Aquac. Res..

[91] Alishahi A., Mirvaghefi A., Tehrani M.R., Farahmand H., Koshio S., Dorkoosh F.A., Elsabee M.Z. (2011). Chitosan nanoparticle to carry vitamin C through the gastrointestinal tract and induce the non-specific immunity system of rainbow trout (*Oncorhynchus mykiss*). Carbohydr. Polym..

[92] Ferosekhan S., Gupta S., Singh A.R., Rather M.A., Kumari R., Kothari D.C., Pal A.K., Jadhao S.B. (2014). RNA-loaded chitosan nanoparticles for enhanced growth, immunostimulation and disease resistance in fish. Curr. Nanosci..

[93] Ramos E.A., Relucio J.L.V., Torres-Villanueva C.A.T. (2005). Gene expression in tilapia following oral delivery of chitosan-encapsulated plasmid DNA incorporated into fish feeds. Marine biotechnol..

[94] Kumar S.R., Ahmed V.P.I., Parameswaran V., Sudhakaran R., Babu V.S., Hameed A.S.S. (2008). Potential use of chitosan nanoparticles for oral delivery of DNA vaccine in Asian sea bass (*Lates calcarifer*) to protect from *Vibrio (Listonella) anguillarum*. Fish Shellfish Immunol..

[95] Li L., Lin S.L., Deng L., Liu Z.G. (2013). Potential use of chitosan nanoparticles for oral delivery of DNA vaccine in black seabream *Acanthopagrus schlegelii* Bleeker to protect from *Vibrio parahaemolyticus*. J. Fish Dis..

[96] Aranaz I., Mengíbar M., Harris R., Paños I., Miralles B., Acosta N., Galed G., Heras Á. (2009). Functional characterization of chitin and chitosan. Curr. Chem. Biol..

[97] Jimenez-Fernandez E., Ruyra A., Roher N., Zuasti E., Infante C., Fernandez-Diaz C. (2014). Nanoparticles as a novel delivery system for vitamin C administration in aquaculture. Aquaculture.

[98] Arbos P., Wirth M., Arangoa M.A., Gaborb F., Irache J.M. (2002). AN as a new polymer for the preparation of ligand-nanoparticle conjugates. J. Control. Release.

[99] Trotta M., Gasco M.R., Morel S. (1989). Release of drugs from oil-water microemulsions. J. Control. Release.

[100] Mezei M., Gulasekharam V. (1980). Liposomes-a selective drug delivery system for the topical route of administration I. Lotion dosage form. Life Sci..

[101] Lieb L.M., Ramachandran C., Egbaria K., Weiner N. (1992). Topical delivery enhancement with multilamellar liposomes into pilosebaceous units: I. *In vitro* evaluation using fluorescent techniques with the hamster ear model. J. Investing. Dermatol..

[102] Honzak L., Šentjurc M. (2000). Development of liposome encapsulated clindamycin for treatment of acne vulgaris. Pflügers Archiv..

[103] Verma D.D., Verma S., Blume G., Fahr A. (2003). Particle size of liposomes influences dermal delivery of substances into skin. Int. J. Pharm..

[104] Knudsen N.O., Jorgensen L., Hansen J., Vermehren C., Frokjaer S., Foged C. (2011). Targeting of liposome-associated calcipotriol to the skin: effect of liposomal membrane fluidity and skin barrier integrity. Int. J. Pharm..

[105] Romoren K., Thu B.J., Smistad G., Evensen O. (2002). Immersion delivery of plasmid DNA I. A study of the potentials of a liposomal delivery system in rainbow trout (*Oncorhynchus mykiss*) fry. J. Control. Release.

[106] Maherani B., Arab-Tehrany E., Mozafari M.R., Gaiani C., Linder M. (2011). Liposomes: A review of manufacturing techniques and targeting strategies. Curr. Nanosci..

[107] Meure L.A., Foster N.R., Dehghani F. (2008). Conventional and dense gas techniques for the production of liposomes: A review. AAPS Pharm. Sci. Tech..

[108] Berger N., Sachse A., Bender J., Schubert R., Brandl M. (2001). Filter extrusion of liposomes using different devices: comparison of liposome size, encapsulation efficiency, and process characteristics. Int. J. Pharm..

[109] Maherani B., Arab-Tehrany E., Kheirolomoom A., Reshetov V., Stebe M.J., Linder M. (2012). Optimization and characterization of liposome formulation by mixture design. The Analyst..

[110] Hope M.J., Bally M.B., Webb G., Cullis P.R. (1985). Production of large unilamellar vesicles by a rapid extrusion procedure—Characterization of size distribution, trapped volume and ability to maintain a membrane-potential. BBA Biomembr..

[111] Macdonald R.C., Macdonald R.I., Menco B.P.M., Takeshita K., Subbarao N.K., Hu L.R. (1991). Small-volume extrusion apparatus for preparation of large, unilamellar vesicles. BBA Biomembr..

[112] Mozafari M.R. (2006). Nanocarrier Technologies: Frontiers of Nanotherapy.

[113] Harikrishnan R., Kim J.S., Balasundaram C., Heo M.S. (2012). Vaccination effect of liposomes entrapped whole cell bacterial vaccine on immune response and disease protection in *Epinephelus bruneus* against *Vibrio harveyi*. Aquaculture.

[114] Irie T., Watarai S., Iwasaki T., Kodama H. (2005). Protection against experimental *Aeromonas salmonicida* infection in carp by oral immunisation with bacterial antigen entrapped liposomes. Fish Shellfish Immunol..

[115] Nakhla A.N., Szalai A.J., Banoub J.H., Keough K.M.W. (1997). Serum anti-LPS antibody production by rainbow trout (*Oncorhynchus mykiss*) in response to the administration of free and liposomally-incorporated LPS from Aeromonas salmonicida. Fish Shellfish Immunol..

[116] Rodgers S. (1990). Immersion vaccination for control of fish furunculosis. Dis. Aqua. Org..

[117] Yasumoto S., Kuzuya Y., Yasuda M., Yoshimura T., Miyazaki T. (2006). Oral immunization of common carp with a liposome vaccine fusing koi herpesvirus antigen. Fish Pathol..

[118] Ruyra A., Cano-Sarabia M., Mackenzie S.A., Maspoch D., Roher N. (2013). A novel liposome-based nanocarrier loaded with an LPS-dsRNA cocktail for fish innate immune system stimulation. PLoS ONE.

[119] Behera T., Swain P. (2011). Antigen adsorbed calcium phosphate nanoparticles stimulate both innate and adaptive immune response in fish, *Labeo rohita* H.. Cell. Immunol..

[120] Faikoh E.N., Hong Y.H., Hu S.Y. (2014). Liposome-encapsulated cinnamaldehyde enhances zebrafish (*Danio rerio*) immunity and survival when challenged with *Vibrio vulnificus* and *Streptococcus agalactiae*. Fish Shellfish Immunol..

[121] Falco A., Barrajón-Catalán E., Menéndez-Gutiérrez M.P., Coll J., Micol V., Estepa A. (2013). Melittin-loaded immunoliposomes against viral surface proteins, a new approach to antiviral therapy. Antivir. Res..

[122] Irie T., Watarai S., Kodama H. (2003). Humoral immune response of carp (*Cyprinus carpio*) induced by oral immunization with liposome-entrapped antigen. Dev. Comp. Immunol..

[123] Rauta P.R., Nayak B. (2015). Parenteral immunization of PLA/PLGA nanoparticle encapsulating outer membrane protein (Omp) from *Aeromonas hydrophila*: Evaluation of immunostimulatory action in *Labeo rohita* (rohu). Fish Shellfish Immunol..

[124] Hølvold L.B., Fredriksen B.N., Bøgwald J., Dalmo R.A. (2013). Transgene and immune gene expression following intramuscular injection of Atlantic salmon (*Salmo salar* L.) with DNA-releasing PLGA nano- and microparticles. Fish Shellfish Immunol..

[125] Tian J., Yu J. (2011). Poly (lactic-co-glycolic acid) nanoparticles as candidate DNA vaccine carrier for oral immunization of Japanese flounder (*Paralichthys olivaceus*) against lymphocystis disease virus. Fish Shellfish Immunol..

[126] Adomako M., St-Hilaire S., Zheng Y., Eley J., Marcum R.D., Sealey W., Donahower B.C., LaPatra S., Sheridan P.P. (2012). Oral DNA vaccination of rainbow trout, *Oncorhynchus mykiss* (Walbaum), against infectious haematopoietic necrosis virus using PLGA [Poly(d,l-Lactic-Co-Glycolic Acid)] nanoparticles. J. Fish Dis..

[127] Fredriksen B.N., Sævareid K., McAuley L., Lane M.E., Bogwald J., Dalmo R.A. (2011). Early immune responses in Atlantic salmon (*Salmo salar* L.) after immunization with PLGA nanoparticles loaded with a model antigen and β-glucan. Vaccine.

[128] Fredriksen B.N., Grip J. (2012). PLGA/PLA micro- and nanoparticle formulations serve as antigen depots and induce elevated humoral responses after immunization of Atlantic salmon (*Salmo salar* L.). Vaccine.

[129] Trapani A., Mandracchia D., Di Franco C., Cordero H., Morcillo P., Comparelli R., Cuesta A., Esteban M.A. (2015). *In vitro* characterization of 6-Coumarin loaded solid lipid nanoparticles and their uptake by immunocompetent fish cells. Coll. Surf..

[130] Jain R.A. (2000). The manufacturing techniques of various drug loaded biodegradable poly (lactide-co-glycolide) (PLGA) devices. Biomaterials.

[131] Danhier F., Ansorena E., Silva J.M., Coco R., Le Breton A., Préat V. (2012). PLGA-based nanoparticles: An overview of biomedical applications. J. Control. Release.

[132] Vert M., Mauduit J., Li S. (1994). Biodegradation of PLA/GA polymers: increasing complexity. Biomaterials.

[133] O’Hagan D.T., Jeffery H., Roberts M., McGee J.P. (1991). Controlled release microparticles for vaccine development. Vaccine.

[134] Panyam J., Labhasetwar V. (2003). Biodegradable nanoparticles for drug and gene delivery to cells and tissue. Adv. Drug Deliv. Rev..

[135] Kapoor D.N., Bhatia A., Kaur R., Sharma R., Kaur G., Dhawan S. (2015). PLGA: A unique polymer for drug delivery. Ther. Deliv..

[136] McCall R.L., Sirianni R.W. (2013). PLGA Nanoparticles Formed by Single- or Double-emulsion with Vitamin E-TPGS. JoVE.

[137] Newman K.D., Elamanchili P., Kwon G.S., Samuel J. (2002). Uptake of poly(d,l-lactic-co-glycolic acid) microspheres by antigen-presenting cells *in vivo*. J. Biomed. Mat. Res..

[138] Lassalle V., Ferreira M.L. (2007). PLA nano- and microparticles for drug delivery: An overview of the methods of preparation. Macromol. Biosci..

[139] Xiao R.Z., Zeng Z., Zhou G.L., Wang J.J., Li F.Z., Wang A.M. (2010). Recent advances in PEG–PLA block copolymer nanoparticles. Int. J. Nanomed..

[140] Iliev D.B., Skjæveland I., Jørgensen J.B. (2013). CpG oligonucleotides bind TLR9 and RRM-Containing proteins in Atlantic Salmon (*Salmo salar*). BMC Immunol..

[141] Alonso M.C., Cano I., Garcia-Rosado E., Castro D., Lamas J., Barja J.L., Borrego J.J., Bergmann S.M. (2005). Isolation of lymphocystis disease virus from sole, *Solea senegalensis* Kaup, and blackspot sea bream, *Pagellus bogaraveo* (Brunnich). J. Fish Dis..

[142] Loomba L., Sekhon B.S. (2015). Calcium Phosphate Nanoparticles and their Biomedical Potential. J. Nanomater. Mol. Nanotechnol..

[143] Guo S., Huang L. (2014). Nanoparticles containing insoluble drug for cancer therapy. Biotechnol. Adv..

[144] De Jesus M.B., Zuhorn I.S. (2015). Solid lipid nanoparticles as nucleic acid delivery system: Properties and molecular mechanisms. J. Control. Release.

[145] Harde H., Das M., Jain S. (2011). Solid lipid nanoparticles: An oral bioavailability enhancer vehicle. Expert Opin. Drug Deliv..

[146] Parhi R., Suresh P. (2012). Preparation and characterization of solid lipid nanoparticles—A review. Curr. Drug Discov. Technol..

[147] Machado A.H.E., Lundberg D., Ribeiro A.J., Veiga F.J., Miguel M.G., Lindman B., Olsson U. (2013). Encapsulation of DNA in macroscopic and nanosized calcium alginate gel particles. Langmuir.

[148] Brudeseth B.E., Wiulsrød R., Fredriksen B.N., Lindmo K., Løkling K.-E., Bordevik M., Steine N., Klevan A., Gravningen K. (2013). Status and future perspectives of vaccines for industrialised fin-fish farming. Fish Shellfish Immunol..

[149] Jovanović B., Palić D. (2012). Immunotoxicology of non-functionalized engineered nanoparticles in aquatic organisms with special emphasis on fish—Review of current knowledge, gap identification, and call for further research. Aqua. Toxicol..

[150] Jovanović B., Whitley E.M., Kimura K., Crumpton A., Palić D. (2015). Titanium dioxide nanoparticles enhance mortality of fish exposed to bacterial pathogens. Environ. Pollut..

